# Comment on ‘Prevalence of sarcopenia in patients with chronic kidney disease: a global systematic review and meta‐analysis’

**DOI:** 10.1002/jcsm.13490

**Published:** 2024-05-15

**Authors:** Xiao‐Ming Zhang, Zaigui Luo, Xiao‐Min Xie, Yunzhi Yang

**Affiliations:** ^1^ Department of Emergency The People's Hospital of Baoan Shenzhen Shenzhen China; ^2^ Guangxi University of Chinese Medicine Nanning China; ^3^ Department of Nursing The People's Hospital of Baoan Shenzhen Shenzhen China

We have recently had the pleasure of delving into an article titled ‘Prevalence of Sarcopenia in Patients with Chronic Kidney Disease: A Global Systematic Review and Meta‐Analysis’ authored by Duarte and their esteemed colleagues.^1^ This meticulously conducted study amalgamated data from 140 research papers, encompassing a staggering 42 041 patients across 25 different countries. The findings revealed a worldwide prevalence of sarcopenia among individuals with chronic kidney disease (CKD) at 24.5% (95% confidence interval [CI]: 20.9–28.3). We were deeply intrigued by the manifold clinical implications that this study has unearthed. Notably, it marks a significant milestone in our comprehension of the impact of sarcopenia within CKD populations. The global perspective it provides serves as a beacon, underscoring the imperative need for standardized screening and management protocols.

Additionally, the authors astutely recommend that future research endeavours shift their focus towards longitudinal studies aimed at unravelling causality alongside interventions that may ameliorate the deleterious effects of sarcopenia in CKD patients. We extend our hearty congratulations to the authors for their admirable work. Nevertheless, we believe certain salient issues warrant meticulous examination to enhance the accuracy and reliability of this research.

Firstly, upon scrutinizing *tab. S2*, it has come to our attention that the authors have provided an overview of the characteristics of the included 140 studies. Notably, we discern that while 115 studies furnished data on the prevalence of sarcopenia among CKD patients, *fig. 2A* illustrates a meta‐analysis involving 114 articles. This discrepancy raises concerns about the potential omission of a study in the meta‐analysis. To rectify this discrepancy, we undertook a re‐analysis of the 115 included studies, yielding a pooled prevalence of sarcopenia among CKD patients closely mirroring Duarte's findings, at 24.4% (95% CI: 21.5–27.4%), as shown in *Figure* *1*.

**Figure 1:**
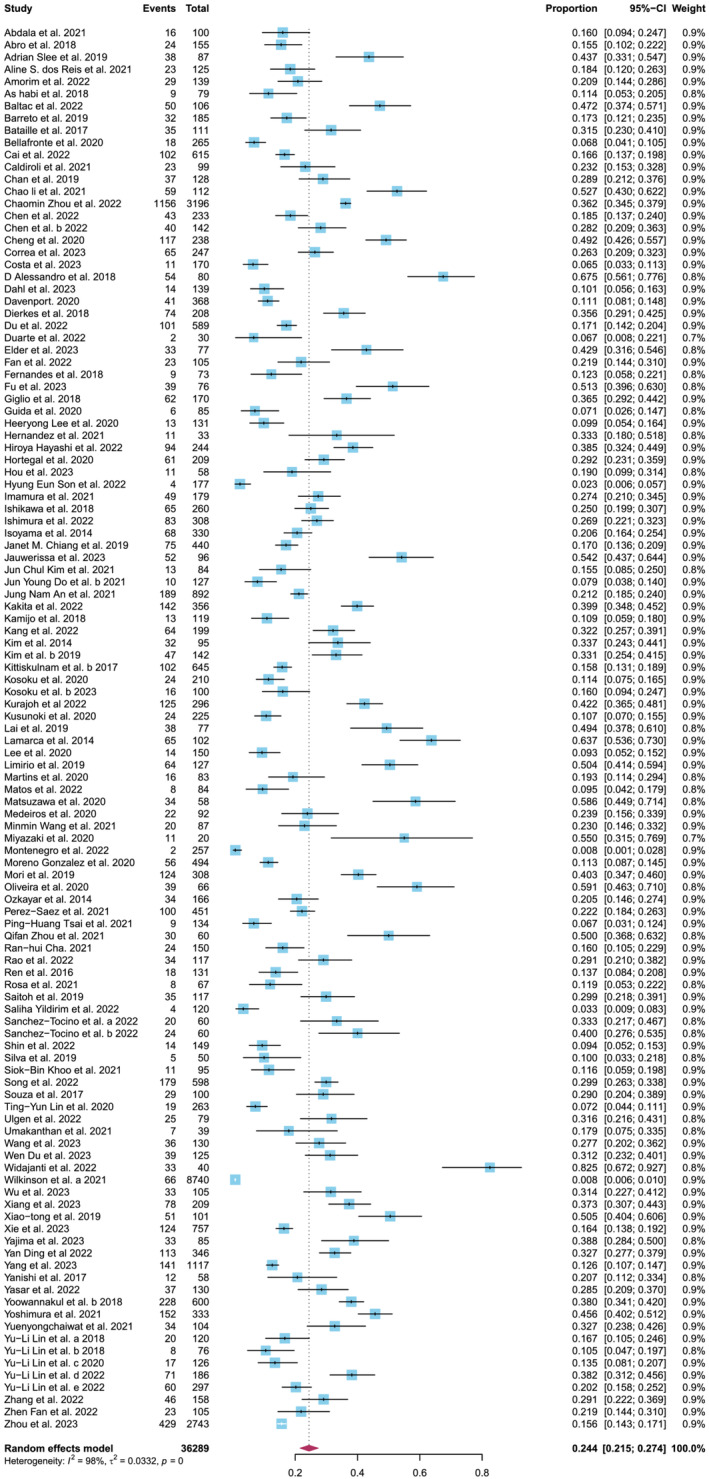
Meta‐analysis for the prevalence of sarcopenia among chronic kidney disease. CI, confidence interval.

Secondly, we advocate for a subgroup analysis based on study design aimed at exploring potential variations in sarcopenia prevalence within this cohort. Our findings indicate that the pooled prevalence of sarcopenia stands at 24.3% (95% CI: 21.0–27.7%) for cross‐sectional studies, 23.8% (95% CI: 16.7–31.8%) for prospective cohort studies and 28.3% (95% CI: 18.3–39.5%) for retrospective cohort studies, with no statistically significant differences observed (*P* = 0.77).

Thirdly, it is worth noting that sarcopenic obesity emerges as a significant concern among CKD patients. While the article states that ‘Five included studies reported the prevalence of sarcopenic obesity’, we have identified six studies in *fig. 4* of Duarte's study. Therefore, we suggest a revision of the statement to accurately reflect this: Six included studies reported the prevalence of sarcopenic obesity. Moreover, given that sarcopenic obesity represents a novel concept in gerontological research,^2^ it would greatly benefit our readers if the authors could provide more detailed information regarding the specific cut‐off values utilized in each included study to define sarcopenic obesity. Inclusion of such data in the supporting information files would enrich the comprehensibility of the research.

Fourthly, considering that sarcopenia tends to escalate with advancing age,^3^ it is reasonable to speculate that individuals aged 65 and older with CKD may exhibit a higher prevalence of sarcopenia compared with their younger counterparts. Our subgroup analysis indeed substantiates this hypothesis, revealing a prevalence of 28% (95% CI: 23–34%) among the older cohort versus 22% (95% CI: 18–25%) among those younger than 65 years old, with a significant difference (*P* = 0.04).

Lastly, adhering to the principles outlined in the PRISMA 2020 checklist, we encourage the authors to conduct sensitivity analyses to assess the robustness of their synthesized results. We believe that such an evaluation would further fortify the reliability of the findings.

In summation, while we commend the authors for their outstanding systematic review and meta‐analysis, characterized by a comprehensive search and rigorous statistical analysis, we urge a careful examination of the identified errors. Furthermore, we advocate for additional subgroup analyses and emphasize the importance of performing sensitivity analyses to fortify the research's credibility.

## Conflict of interest statement

None to disclose.
